# High-speed Beveled Tip Versus Standard Tip Vitrectomy Probe: A Prospective Randomized Clinical Trial

**DOI:** 10.18502/jovr.v18i4.14552

**Published:** 2023-11-30

**Authors:** Shriji Patel, Archana Nair, Kenneth Taubenslag, Kurt Scavelli, Paul Mallory, Tomas Moreno, Rishabh Date, Heather Tamez

**Affiliations:** ^1^Vanderbilt University Medical Center, Department of Ophthalmology, Nashville, TN, USA; ^2^University of Maryland, Department of Ophthalmology, Baltimore, MD, USA; ^3^Florida Retina Institute, Jacksonville, FL, USA; ^4^NJ Retina, Wayne, NJ, USA; ^5^Retina Care Center, Baltimore, MD, USA

**Keywords:** Retina; Surgery, Small-gauge Vitrectomy, Advanced UltraVit, Beveled Probe

## Abstract

**Purpose:**

To compare the efficiency of the advanced ultravit beveled vitrector probe (10,000 cuts per minute) to the current standard ultravit highspeed (7500 cuts per minute) vitrector probe.

**Methods:**

A prospective, randomized controlled trial was conducted on patients undergoing routine vitrectomy surgery for epiretinal membrane, full-thickness macular hole, and vitreous opacities. Patients were randomly assigned to undergo PPV with the ultravit highspeed probe (Probe 1) or the advanced ultravit beveled probe (Probe 2). The main outcome measure was time to completion of core vitrectomy and vitreous base shave.

**Results:**

Forty patients were enrolled in this study, 20 in each cohort. The average time to completion of core vitrectomy was 10.4 +/- 1.8 min in the Probe 1 cohort compared to 9.7 +/- 2 min in the Probe 2 cohort (*P* = 0.21). The average time to completion of vitreous base shave was 9.6 +/- 2.7 min in the Probe 1 cohort compared to 9.4 +/- 1.8 min in the Probe 2 cohort (*P* = 0.39).

**Conclusion:**

In the current study, the advanced ultravit beveled probe was noninferior to the ultravit highspeed vitrectomy probe when looking at the time to completion of core vitrectomy and vitreous base shave. The increased cut rate did not affect the efficiency of vitreous removal.

##  INTRODUCTION

Since the initial description of modern vitrectomy surgery by Machemer^[1]^, significant advancements have been made in the field of vitreoretinal surgery. These developments include smaller gauge instrumentation, more efficient vitrectomy systems with higher cut rates, and improved wide-field posterior segment visualization. Newer designs of vitrectomy probes have maximized duty cycle performance while improving flow and tissue interaction. As the instrumentation has evolved, the capabilities of surgeons have expanded as have the indications for vitrectomy surgery.

Streamlined, smaller gauge instrumentation with high cut rates can help reduce surgical times as well as postoperative pain and inflammation compared to 20-gauge vitrectomy systems.^[2]^ They also facilitate vitreous removal while minimizing the sphere of influence and risk of inadvertent retinal traction.^[3]^ In recent years, high-speed vitrectomy probes have gained in popularity, especially given their favorable safety profile. Vitrectomy probes are now commercially available with cut rates of up to 20,000 cuts per minute (cpm). However, increased cut rates in smaller gauge vitrectomy systems can adversely affect flow rates and vitreous removal since spring-opening pneumatic closure vitrectomy probes lend themselves to a reduced duty cycle at high speeds.^[4,5]^ Dual-pneumatic vitrectomy probes have the ability to overcome these limitations by modulating the duty cycle.

**Figure 1:**
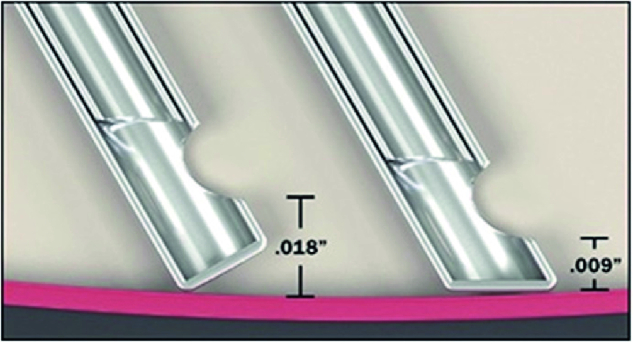
Comparison of port opening between the standard tip vitrectomy probe (left) and beveled tip vitrectomy probe (right). The distance from the vitrector mouth opening to the tissue plane is reduced in half by introduction of the beveled tip.

The advanced ultravit beveled probe (Alcon, Fort Worth, TX) is a dual-pneumatic vitrectomy probe with a maximum cut rate of 10,000 cpm. The beveled-tip design allows the opening of the vitrector to gain closer approximation when manipulating tissue planes compared to the standard vitrector probe design [see Figure 1].

The beveled opening gives surgeons enhanced maneuverability in tight tissue planes and increases the functionality of the vitrectomy probe. There is limited data regarding the effect of this probe design and cut rate on the efficiency of vitreous removal. The authors set out to compare the efficiency of this probe compared to the standard ultravit highspeed probe, which is similarly a dual-pneumatic actuation probe but features a maximum cut rate of 7500 cpm.

##  METHODS

This study was approved by the Vanderbilt University (USA) Institutional Review Board (IRB#190728) and complied with the tenets of the Declaration of Helsinki. Written informed consent was obtained by all study participants.

The authors conducted a prospective, randomized controlled trial (ClinicalTrials.gov Identifier NCT04076072) comparing the efficiency of the new advanced ultravit beveled to the current standard (non-beveled) vitrector probe, the ultravit highspeed. Patients were considered for study inclusion if they were 18 years or older undergoing routine vitrectomy surgery for vitreous opacities of visual significance, nonclearing vitreous hemorrhage, vitreomacular traction syndrome, full-thickness macular hole or epiretinal membrane. Patients were excluded if they had previous intraocular surgery other than noncomplex cataract extraction. To reduce surgical time variability, any complex surgical indications (e.g., rhegmatogenous retinal detachment, tractional retinal detachment) were excluded.

On the day of surgery, informed consent was obtained, and patients were randomized by a masked study coordinator for undergoing vitrectomy surgery with the advanced ultravit beveled or the ultravit highspeed vitrectomy probe. A total of 40 patients were enrolled, 20 in each cohort. All surgeries were performed by a single surgeon (SP) with the assistance of a vitreoretinal fellow. All vitrectomies were performed using 25-gauge instrumentation and featured uniform constellation (Alcon, Fort Worth, Tx) settings including infusion pressure of 30 mmHg. For core vitrectomy, 650 mmHg vacuum pressure was used; however, this vacuum pressure was reduced to 400 mmHg for the vitreous base shave. The BIOM (Oculus Surgical, Port St. Lucie, Fl) non-contact wide-angle visualization system was used for all vitrectomies.

The main outcome measures were the time required for completion of core vitrectomy (minutes) and completion of the vitreous base shave (minutes). Time for these outcome measures were recorded using a masked timer in the room observing the surgery in real time. A study coordinator reviewed the electronic medical record and noted any intraoperative complications (e.g., iatrogenic retinal breaks, intraocular bleeding, retinal detachment) and postoperative complications.

Postoperative visits were conducted on day 1, week 1, month 1, and month 3 following surgery. Standard assessments including best-corrected visual acuity and intraocular pressure were conducted by a masked ophthalmic technician. Slit-lamp examination and indirect ophthalmoscopy were conducted at each visit along with evaluation for any adverse events.

### Sample Size Calculation

The surgeons, using existing internal data, assumed an average time for core vitrectomy completion of 12 +/- 3 min. Therefore, a sample size of at least 32 eyes would be required to adequately assess for a clinically significant difference between the two groups with 80% power at a 95% confidence interval. A p-value of < 0.05 was deemed to be statistically significant.

### Statistical Analysis

All data were securely recorded and stored by a masked study coordinator in REDCap (Vanderbilt University). Descriptive statistics were employed for this analysis using R 4.0.5 (R Foundation for Statistical Computing, Vienna, Austria) with two ‐ sided significance testing and statistical significance set at a level of 0.05.

##  RESULTS

Forty patients were enrolled in the study, with 20 patients undergoing surgery with the Ultravit Highspeed 7500 cpm vitrectomy probe (Probe 1) and 20 patients undergoing surgery with the dvanced ultravit beveled vitrectomy probe (Probe 2). Surgical indications included epiretinal membrane, vitreous opacities, and full-thickness macular hole [Table 1]. The average age of patients undergoing surgery was 64.2 +/- 6 years; 31/40 patients were pseudophakic at the time of surgery. The average preoperative best-corrected visual acuity was 20/54. The average postoperative best corrected visual acuity at three-month follow-up was 20/30.

**Table 1:** Surgical Indication.


	orangeProbe 1	orangeProbe 2
**Surgical Indication**	
Epiretinal membrane	2	3
Vitreous opacities	10	13
Full-thickness macular hole	8	4
	
	

### Vitrectomy Time

Average time to completion of core vitrectomy was 10.4 +/- 1.8 min in the Probe 1 cohort compared to 9.7 +/- 2 min in the Probe 2 cohort (*P* = 0.21). Average time to completion of vitreous base shave was 9.6 +/- 2.7 min in the Probe 1 cohort compared to 9.4 +/- 1.8 min in the Probe 2 cohort (*P *= 0.39). No significant differences existed in the time to completion of vitrectomy based on surgical indication.

### Safety 

No intraoperative complications were noted in either cohort, including no iatrogenic retinal breaks, retinal detachment, or inadvertent lens trauma. No significant adverse events were noted in the postoperative follow-up period. One eye in the Probe 2 cohort experienced late macular hole reopening.

##  DISCUSSION

In the current study, the advanced ultravit beveled 10,000 cpm vitrectomy probe was noninferior to the ultravit highspeed 7500 cpm vitrectomy probe when looking at time to completion of core vitrectomy and vitreous base shave. The findings of this study suggest that the increased cut rate associated with the advanced ultravit beveled probe did not negatively impact efficiency of vitreous removal. Additionally, the beveled tip, which is meant to create greater access to the vitreous at the retina interface, did not negatively alter vitreous removal speeds.

Compared to previous iterations of vitrectomy systems, newer instrumentation emphasizes smaller gauge to minimize surgical trauma; 23-, 25-, and 27-gauge instrumentations have a smaller sphere of influence compared to older 20-gauge systems, limiting unintended tissue interactions. At the same time, smaller gauge vitrectomy can reduce fluid flow. The internal diameter of vitrector probes varies from 0.52 mm for 20g, 0.36–0.39 mm for 23g, 0.26–0.29 mm for 25g, and 0.20 mm for 27g systems.^[6]^ Simultaneously, cut rates have evolved from 1 cut per second to 20,000 cuts per minute. Higher cut rates reduce the `bite' size and thus the effective viscosity of non-Newtonian fluids such as vitreous. These limitations are counteracted by higher aspiration vacuums and duty cycle modulations afforded by more advanced vitrectomy systems. Considering all these factors, there is an ongoing debate on the effect of newer small-guage vitrectomy systems on the efficiency of vitreous removal. Our data demonstrated the noninferiority of the increased cut rate in a clinical setting.

Our study has several important limitations. The main outcome measures (time to completion of vitrectomy and vitreous base shave) have some inherent level of subjectivity. Masking of the surgeon could not be achieved in this setting given the difference in appearance of the vitrectomy probes and this is an important potential confounder. However, masking of the trained timer viewing the surgeon was obtained. Different surgeon experiences can also affect the efficiency of vitreous removal. The protocol called for a single-surgeon and uniform vitrectomy settings (e.g., gauge, infusion pressure, and vacuum pressure) to account for this potential variability. All cases in this setting were fellow assisted, which was critical for the scleral depression during the vitreous base shave. The applicability of this data may be hindered outside the setting where a skilled surgical assistant is present.

In summary, the advanced ultravit beveled vitrectomy probe was noninferior to the ultravit highspeed vitrectomy probe when looking at time to completion of core vitrectomy and vitreous base shave. Further research should concentrate on the potential benefits of the bevel-tip vitrectomy probe and increasingly faster vitreous cut rates.

##  Funding

This work was supported by an Alcon Investigator-Initiated Grant (IIT# 46218371). The funding organization had no role in the design or conduct of this research or the writing of this manuscript.

##  Conflicts of Interest 

None
